# Bypass Procedure Performed in the Field of a Decompressive Craniectomy in the Case of an MCA Dissecting Aneurysm: Case Report and Review of the Literature

**DOI:** 10.3390/brainsci11010029

**Published:** 2020-12-29

**Authors:** Robert Bartoš, Jan Lodin, Aleš Hejčl, Ivan Humhej, Ingrid Concepción, Filip Cihlář, Martin Sameš

**Affiliations:** 1 Department of Neurosurgery, J. E. Purkyne University, Masaryk Hospital, 401 13 Ústí nad Labem, Czech Republic; robert.bartos@kzcr.eu (R.B.); jan_lodin@hotmail.com (J.L.); ivan.humhej@kzcr.eu (I.H.); ingrid1585.ic@gmail.com (I.C.); martin.sames@kzcr.eu (M.S.); 2 Institute of Anatomy, 1st Medical Faculty, Charles University, 128 00 Prague, Czech Republic; 3 International Clinical Research Center, St. Anne’s University Hospital, 656 91 Brno, Czech Republic; 4 Institute of Experimental Medicine, Academy of Sciences of the Czech Republic, 142 20 Prague, Czech Republic; 5 Department of Neurosurgery, Complejo Hospitalario Metropolitano Dr. Arnulfo Arias Madrid, Panama 07096, Panama; 6 Department of Radiology, J. E. Purkyne University, Masaryk Hospital, 401 13 Ústí nad Labem, Czech Republic; filip.cihlar@kzcr.eu

**Keywords:** aneurysm, extra-intracranial (EC-IC) bypass, decompressive craniectomy, superficial temporal artery (STA)

## Abstract

Treatment of complex aneurysms often requires additional surgical tools including the use of the extra-intracranial (EC-IC) bypass. The following report depicts the utilization of the EC-IC bypass in treating a dissecting aneurysm several hours after a salvage emergent evacuation of an acute subdural hematoma via decompressive craniectomy (DC). Preserving the superficial temporal artery during the DC provided a donor artery for the bypass surgery.

## 1. Introduction

Utilization of the extra-intracranial (EC-IC) bypass in the treatment of cerebral aneurysms is an essential component of a surgeon’s armamentarium and as such has been described in several case series by master surgeons, who focus on the most difficult of cases [1,2,3,4,5,6,7,8]. The following report depicts the utilization of this procedure in the case of a young patient admitted with a severe neurological deficit. The patient underwent a decompressive craniectomy (DC), evacuation of an acute subdural hematoma and 8 h later, in a separate procedure, the trapping of an unclippable aneurysm via an EC-IC bypass, which preserved flow in the middle temporal branch of the middle cerebral artery (MCA). Our case is unique in that the vessel donor was a superficial temporal artery (STA) branch, which was dissected from the supragaleal space within the skin flap from the DC.

## 2. Case Report

**Figure 1:**
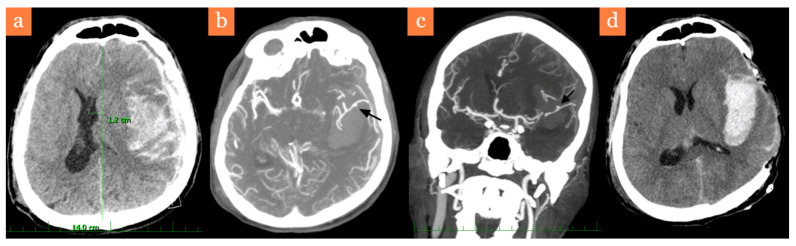
Graphic depiction of the case before the second surgery. (**a**) CT performed immediately after admission, showing a severe midline shift of 12 mm, (**b**,**c**) axial and coronal CT angiography (CTA) views depicting a large intracerebral hematoma in the sylvian fissure and a broad base M3 aneurysm, (**d**) postoperative CT after decompressive craniectomy (DC), without complications with midline shift regression and the intact intracerebral hematoma.

A 43-year-old male was admitted in July 2019 to the emergency department after being found unconscious in the street with anisocoria, severe right-sided hemiparesis with a Glasgow Coma Scale of 6 (E1V2M3). His initial clinical condition suggested head trauma due to alcohol intoxication. A head CT was performed at the local hospital (Figure 1a), and the patient was sedated, intubated and transported to our hospital in order to perform a DC. The neurosurgeon on duty requested a preoperative CT angiography (CTA), which showed a suspect dissecting aneurysm of the M3 segment of the middle temporal branch of the left MCA (Figure 1b,c). A DC was urgently performed in the evening and after consulting his superiors, the surgeon on duty did not immediately treat the aneurysm. However, he carefully preserved the STA within the supragaleal space of the skin flap. Early next morning, a postoperative CT was performed with acceptable results of midline shift regression (Figure 1d).

**Figure 2:**
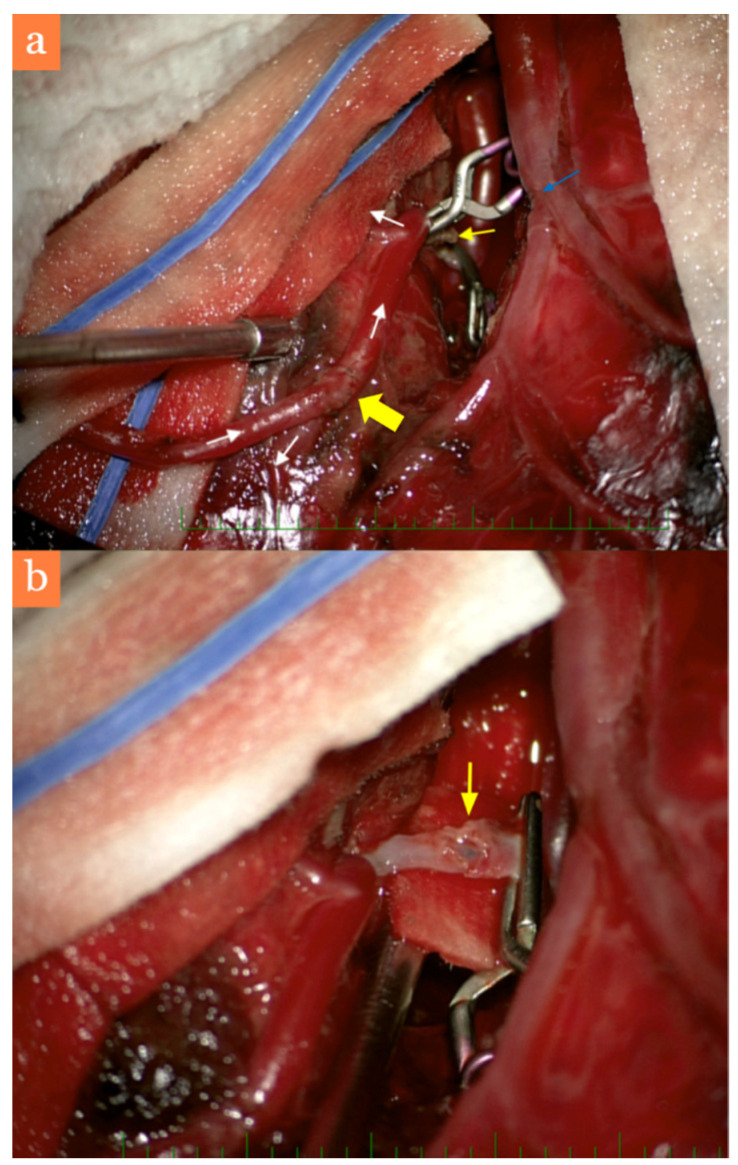
Intraoperative situations (**a**) final aneurysm treatment: extra-intracranial (EC-IC) (superficial temporal artery–middle cerebral artery (STA-MCA) M4) bypass (white arrows showing the direction of flow, site of the anatomosis has wide yellow arrow), aneurysm trapping (thin yellow arrow), sylvian fissure dissection with suturing of a superficial sylvian vein branch (blue arrow), primary insult of the cerebral parenchyma via an acute subdural and intracerebral hematoma, (**b**) detailed dissection of the M3 arterial wall.

However, the patient was hemodynamically unstable, requiring continuous vasopressor support and it was impossible to obtain a valid neurological exam. Eight hours after the initial surgery, a second procedure was scheduled, aiming to treat the dissecting aneurysm. After skin flap eversion and durotomy, the neurosurgeon (first author) preferred dissection of the arterial donor from the skin flap and performed a protective bypass on the distal M4 segment of the middle temporal branch of the MCA (Figure 2a). The reason for this was that performing dissection in the area of the aneurysm was very risky due to adhering coagula. Furthermore, it was uncertain that a direct reconstruction of the artery, which most likely supplied the superior temporal gyrus, would be possible. Patency of the bypass was verified via a transcranial Doppler (TCD), after which dissection of the proximal portion sylvian fissure was initiated, with necessary cutting a superficial sylvian vein branch followed by its suture (Figure 2a). After placement of a temporary clip on the proximal M3 and evacuation of coagula surrounding the aneurysm, the surgeon verified a defect of the arterial wall without the presence of an aneurysmal sac—a true dissecting pseudoaneurysm (Figure 2b). In this case, we considered trapping a short portion of the mother vessel to be safer than direct reconstruction of the arterial wall.

**Figure 3:**
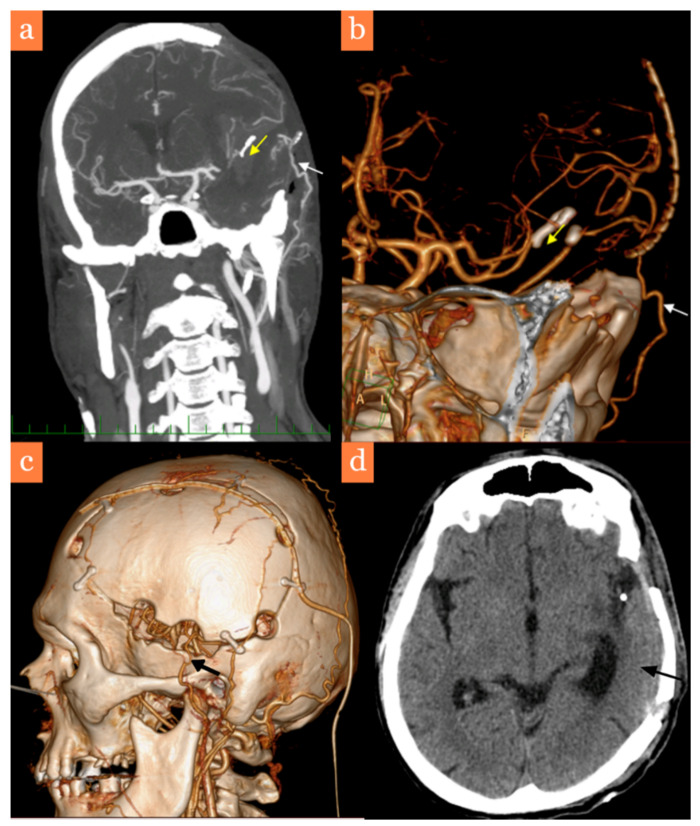
Graphic visualization after the second surgery. (**a**,**b**) CTA on the first postoperative day showing the trapped pseudoaneurysm (yellow arrow) and the EC-IC bypass (white arrow), (**c**) 3D side view after cranioplasty with an autologous bone flap with a CTA demonstrating the patent EC-IC bypass (black arrow), (**d**) CT after cranioplasty showing resorption of the remaining intracerebral hematoma and the ischemia-free temporal lobe (black arrow).

CTA was performed the next day and demonstrated good patency of the bypass, successful trapping of the pseudoaneurysm and sufficient collateral flow (Figure 3a,b). In the postoperative period, the patient required milrinone-mediated pharmacological angioplasty several times, due to moderate vasospasms. Surprisingly, the bypass itself was spared of vasospasms based on TCD measurements (insonation depth of 20–25 mm in the temporal region).

After vasospasms receded, the patient was transferred to his local intensive care unit on the 17th day after the second surgical procedure. Clinically, the patient had a tracheostomy, a moderate right-sided hemiparesis (4/5) and was compliant to vocal commands. Two months later, he was readmitted to our neurosurgical department in order to perform a cranioplasty, which was technically challenging as careful dissection was required to identify and preserve the EC-IC bypass (Figure 3c). A follow-up CT did not reveal any ischemic lesions in the patient’s left temporal lobe (Figure 3d). The patient was then transferred to a rehabilitation department without a tracheostomy, without a hemiparesis and with residual moderate aphasia (modified Rankin scale, mRS3).

As this is a case report, approval of the ethics committee was not necessary for this paper. The participant has signed the written informed consent.

## 3. Discussion

**Figure 4:**
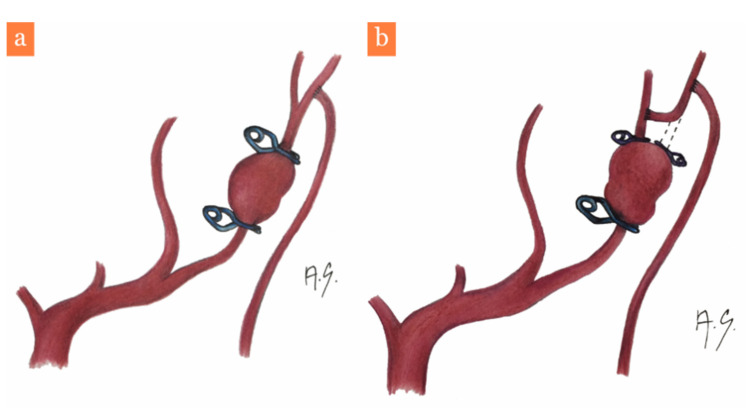
Artistic depiction of the bypass (drawn by Alena Sejkorová). (**a**) Our utilized STA-MCA M4 low flow bypass and M3 aneurysm trapping, (**b**) potential use of the STA-MCA M4 bypass along with reimplantation of the proximal M3 segment into the second branch in cases of two MCA branches exiting the aneurysm, the parietal M2 trunk and its branches are not involved.

Treatment of complex cerebral aneurysms via MCA bypass reconstructions has been recently presented by Lawton et al. in a paper containing 30 patients [9]. Furthermore, the authors offer a decision-making algorithm, which specifies the bypass a surgeon should perform in a set situation. In cases of postbifurcation aneurysms, they suggest trapping the aneurysm with a reanastomosis, reimplantation, an in situ side-to-side bypass or an interposed graft. Only in cases of distal insular aneurysms as a last resort, do they suggest performing a low flow STA-MCA bypass followed by proximal occlusion of the MCA mother vessel. The series is published by an institution renowned for expertise in neurovascular surgery, however, even so, merely 2.1% of the 1426 treated MCA aneurysms over 17 years were selected. This is the equivalent of less than two MCA aneurysms treated with a bypass reconstruction technique every year. Furthermore, only eight patients presented with ruptured aneurysms, 12 patients had a focal neurological deficit and the remaining were treated electively. Bypass patency was recorded to be 90% and 27 patients were in stationary or improved clinical condition. Prior to this paper, the Helsinki group [10] published results of 24 patients with complex MCA aneurysms operated over 14 years. In this group, more EC-IC bypasses were performed (21) than in Lawton et al.’s series, where only four intra-intracranial bypasses were performed. All reported cases were complex aneurysms of large sizes, and only three aneurysms (12%) were smaller than 20 mm. Subarachnoid hemorrhage was the presenting symptom in only two cases (8%), thus the operating surgeons had sufficient time for preoperative preparation, including the utilization of the Excimer Laser Assisted Non-occlusive Anastomosis (ELANA) technique in several cases. In two cases (8%), occlusion of the bypass occurred in the early postoperative period. One patient died in the perioperative period due to perilesional edema and hemorrhage progression (4%) and six patients manifested perioperative ischemic strokes, from which only one patient fully recovered. The high difficulty of these cases is demonstrated by the percentage of permanent surgical morbidity, which was reported to be 21% in this paper. The most high-risk cases tend to be aneurysms of the M1 segment of the MCA, as reconstructions in this area are complicated by the presence of lenticulostriate perforators. It is likely that a general neurosurgeon is confronted with such a situation only once in his/her lifetime and it is vital that he/she is technically prepared to face it. In our opinion, for cases of ruptured postbifurcation MCA aneurysms, it is simpler to perform a direct STA-MCA M4 bypass, utilizing a superficial MCA branch (Figure 4a) as a means of preserving perfusion of the mother vessel territory. In order to identify the correct artery branch as the bypass acceptor, the indocyanine green (ICG) “flash test” can be utilized. The bypass suture is technically simpler on the cerebral surface as opposed to the depths of the sylvian fissure, especially with cerebral edema complicating the surgical approach. We also believe it is superior to a side-to-side bypass or reimplantation, as it does not carry a risk of occluding a second viable vessel. In our case, the operating surgeon decided that reimplantation of the affected artery would be more complicated with a higher chance of vessel occlusion compared to an isolated STA-MCA bypass (Figure 3b). We would consider combining the STA-MCA bypass with reimplantation of the affected artery in the case of a distal M3 aneurysm from which two MCA branches originate. The STA-MCA bypass would then perfuse both branches (Figure 4b) in the same way, as shown by Lawton et al. in their excellent surgical video [11]. This video also accentuates the necessity of delicate microsurgical manipulation with the anastomosis during temporary occlusion of the vessel. A described means of decreasing the risk of perioperative anastomosis occlusion is the intravenous application of eptifibatide, whose safety of use is described by Stambolija et al. in two case reports [12]. In our case, we did not administer any antiaggregant or anticoagulant therapy.

Distal MCA aneurysms can be saccular, fusiform, mycotic or dissecting, as in our case. A similar philosophy and technique to ours is presented by Japanese authors, who consider trapping the aneurysm along with an STA-MCA bypass to be the primary option in cases such as ours. Saito et al. [13] present the case of an unclippable non-dissecting ruptured saccular aneurysm of the central MCA branch and Sakamoto et al. [14] describe the case of a dissecting aneurysm of the M3 MCA segment, both of which were treated using a technique similar to ours. Nakahara et al. [15] depict the complexity in treating distal mycotic aneurysms in five cases. Their approach consists of endovascular coiling/trapping of the aneurysm via occlusion of the mother vessel following a balloon occlusion test. In cases of rupture with a hematoma demonstrating significant mass effect, a surgical revision is indicated to evacuate the hematoma and either clip or trap the aneurysm. This was performed in two of their cases, both of which had a primary diagnosis of sepsis or endocarditis, which was immediately treated with intravenous antibiotics. No subsequent bypasses were performed. Qian et al. [16] describe yet another bypass method utilizing an STA intergraft in the case of mycotic aneurysm of the M3 MCA segment. However, their case was more complicated than ours, requiring two end-to-end anastomoses. Our case differs from the abovementioned cases in that it was performed in the terrain of a DC, utilizing the STA from the everted skin flap as the bypass donor. However, it has to be said that endovascular techniques are rapidly advancing, with Aljuboori et al. [17] recently describing the treatment of a traumatic pseudoaneurysm caused by a gunshot wound, via the Pipeline Flex Embolization Device. In our case, an endovascular treatment strategy would have been possible by occluding the mother vessel. Due to the fact that the artery diameter was merely 1.6 mm, we do not believe an endovascular reconstructive procedure utilizing a stent would be viable option. Implantation of a flow diverter would have carried a high risk of hemorrhagic complications due to the necessary administration of dual antiaggregant therapy.

## 4. Conclusions

In cases requiring DC as a lifesaving surgical procedure, we advocate preserving the STA within the skin flap for potential future EC-IC reconstructions, which may be necessary due to the presence of an undiagnosed vascular lesion, such as a dissecting post-bifurcation MCA aneurysm. In the case above, preserving the STA allowed us to trap the aneurysm and occlude the mother vessel, while preserving flow to potentially eloquent cerebral cortex. Due to the fact that these cases are extremely rare, we prefer trapping the aneurysm and performing an STA-MCA bypass, which is technically simpler than intracranial vascular reconstructions. We believe this is accentuated when performing the procedure in the terrain of a DC with cerebral edema. This is surely a different situation compared to performing the procedure in an elective setting.

## Data Availability

No data available.
